# Expression Profile of Genes Regulating Steroid Biosynthesis and Metabolism in Human Ovarian Granulosa Cells—A Primary Culture Approach

**DOI:** 10.3390/ijms18122673

**Published:** 2017-12-09

**Authors:** Wiesława Kranc, Maciej Brązert, Katarzyna Ożegowska, Mariusz J. Nawrocki, Joanna Budna, Piotr Celichowski, Marta Dyszkiewicz-Konwińska, Maurycy Jankowski, Michal Jeseta, Leszek Pawelczyk, Małgorzata Bruska, Michał Nowicki, Maciej Zabel, Bartosz Kempisty

**Affiliations:** 1 Department of Anatomy, Poznan University of Medical Sciences, 60-781 Poznan, Poland; wkranc@ump.edu.pl (W.K.); mjnawrocki@ump.edu.pl (M.J.N.); mdyszkiewicz@ump.edu.pl (M.D.-K.); m.jankowski.14@aberdeen.ac.uk (M.J.); mbruska@ump.edu.pl (M.B.); 2 Division of Infertility and Reproductive Endocrinology, Department of Gynecology, Obstetrics and Gynecological Oncology, Poznan University of Medical Sciences, 60-101 Poznan, Poland; maciejbrazert@ump.edu.pl (M.B.); k.ozegowska@gmail.com (K.O.); pawelczyk.leszek@ump.edu.pl (L.P.); 3 Department of Histology and Embryology, Poznan University of Medical Sciences, 60-781 Poznan, Poland; joanna.budna@wp.pl (J.B.); pcelichowski@ump.edu.pl (P.C.); mnowicki@ump.edu.pl (M.N.); mazab@ump.edu.pl (M.Z.); 4 Department of Biomaterials and Experimental Dentistry, Poznan University of Medical Sciences, 60-812 Poznan, Poland; 5 Department of Obstetrics and Gynecology, University Hospital and Masaryk University, 625 00 Brno, Czech Republic; jeseta@gmail.com; 6 Department of Histology and Embryology, Wroclaw Medical University, 50-368 Wroclaw, Poland

**Keywords:** human, granulosa cells, in vitro culture (IVC), steroid biosynthesis

## Abstract

Because of the deep involvement of granulosa cells in the processes surrounding the cycles of menstruation and reproduction, there is a great need for a deeper understanding of the ways in which they function during the various stages of those cycles. One of the main ways in which the granulosa cells influence the numerous sex associated processes is hormonal interaction. Expression of steroid sex hormones influences a range of both primary and secondary sexual characteristics, as well as regulate the processes of oogenesis, folliculogenesis, ovulation, and pregnancy. Understanding of the exact molecular mechanisms underlying those processes could not only provide us with deep insight into the regulation of the reproductive cycle, but also create new clinical advantages in detection and treatment of various diseases associated with sex hormone abnormalities. We have used the microarray approach validated by RT-qPCR, to analyze the patterns of gene expression in primary cultures of human granulosa cells at days 1, 7, 15, and 30 of said cultures. We have especially focused on genes belonging to ontology groups associated with steroid biosynthesis and metabolism, namely “Regulation of steroid biosynthesis process” and “Regulation of steroid metabolic process”. Eleven genes have been chosen, as they exhibited major change under a culture condition. Out of those, ten genes, namely *STAR, SCAP, POR, SREBF1, GFI1, SEC14L2, STARD4, INSIG1, DHCR7*, and *IL1B*, belong to both groups. Patterns of expression of those genes were analyzed, along with brief description of their functions. That analysis helped us achieve a better understanding of the exact molecular processes underlying steroid biosynthesis and metabolism in human granulosa cells.

## 1. Introduction

The increase of knowledge about the processes underlining the development of human gametes have brought into light the unique interactions between the tissues involved in that process. Granulosa cells, being a part of almost every stage of folliculo and oogenesis, are deeply involved in storage and maturation of the oocytes, expressing a range of reciprocal interactions with the female gamete [1,2,3]. Additionally, they play a major role in synthesis, expression, and metabolism of a range of hormones, that not only function in maintenance of gamete maturation, regulation of ovulation, and sustenance of pregnancy, but also perform a wide range of secondary functions defining almost every aspect of physiology associated with reproduction [4]. Because of the fact that the mammalian female reproduction cycle is a highly dynamic process, all the processes involved in its regulation and maintenance are extremely complex. Understanding of the exact molecular mechanisms underlining the processes that granulosa cells are a part of directly or indirectly, through endocrinal regulation, not only broadens the knowledge about one of the most essential processes in the mammalian life cycle, but also opens the way for a search for potential clinical possibilities [2,5]. From advances in assisted reproduction, to uncovering the exact mechanism that underline various diseases associated with both the reproduction and hormonal abnormalities, the basis of knowledge that would describe the exact cellular processes that underline those phenomena lies in the patterns of genetic expression associated with all the tissues involved [6,7,8]. In addition, while the functional analyses of those processes have been long performed and provided us with wide understanding of the ways in which granulosa cells function both by themselves and in relation to the developing oocytes, the knowledge about their exact molecular basis is relatively small [9].

By employing the in vitro cultures of human granulosa cells, we aim to analyze and describe changes in the transcriptome during the long term culture. As regulation of steroid sex hormone synthesis, expression, and metabolism is one of the major functions of the granulosa, we have used microarray assays, together with RT-qPCR validation, to analyze the expression patterns of genes involved in the “Regulation of steroid biosynthesis process” and “Regulation of steroid metabolic process”. The genes we aim to identify might later serve for markers for the processes, both normal and pathophysiological, occurring through the whole reproductive cycle, while the expression patterns that they exhibit, together with their mutual relations, might serve as factors to better understand the ways in which granulosa cells behave in vitro and possibly in vivo.

## 2. Results

Whole transcriptome profiling by Affymetrix microarray allowed us to analyze the expression profile of genes regulating steroid biosynthesis and metabolism in human ovarian granulosa cells in primary culture. By Affymetrix^®^ Human HgU 219 Array (Affymetrix, Santa Clara, CA, USA), we examined the expression of 22,480 transcripts. Genes with fold change higher than |2| and with a corrected *p* value lower than 0.05 were considered as differentially expressed. This set of genes consisted of 2278 different transcripts. The first detailed analysis based on the gene ontology biological process (GO BP) allowed for the identification of differentially expressed genes belonging to the significantly enriched GO BP terms.

**Figure 1:**
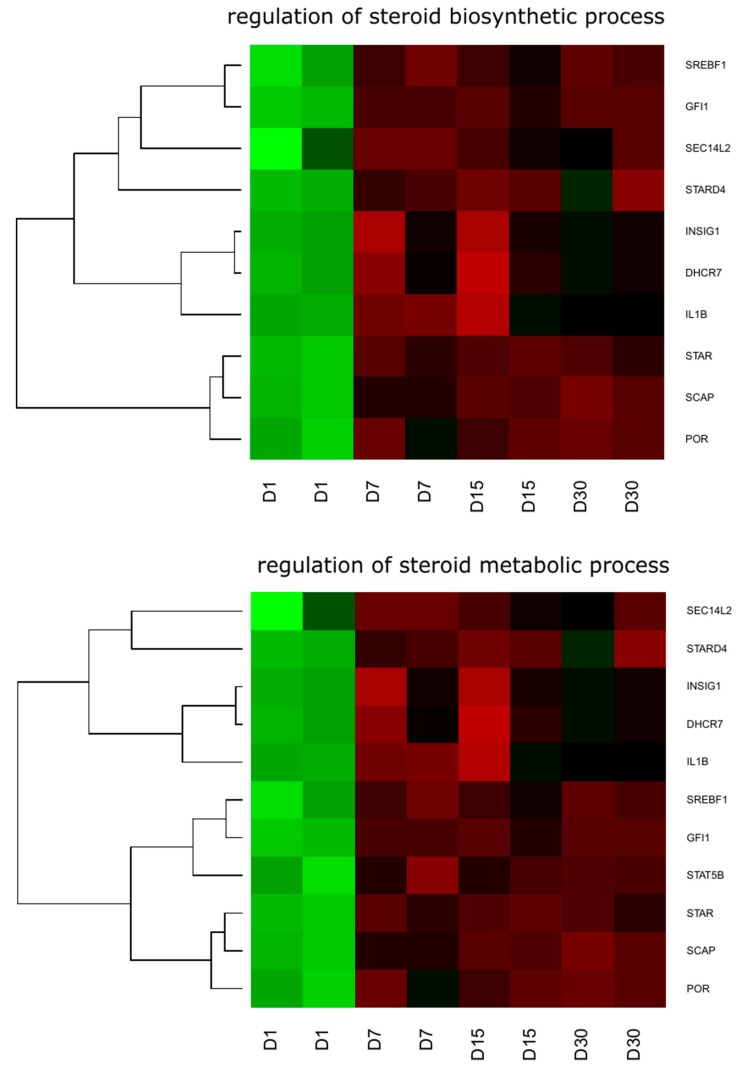
Heat map representation of differentially expressed genes belonging to the “regulation of steroid biosynthetic process” and “regulation of steroid metabolic process” gene ontology biological process (GO BP) terms. Arbitrary signal intensity acquired from microarray analysis is represented by colors (green, higher; red, lower expression). Log2 signal intensity values for any single gene were resized to Row Z-Score scale (from −2, the lowest expression to +2, the highest expression, for a single gene). D: Day of Culture.

DAVID (Database for Annotation, Visualization and Integrated Discovery) software (Leidos Biomedical Research, Inc., National Cancer Institute, Frederick, MD, USA) was used for extraction of the genes belonging to the analyzed GO BP terms. Up and down regulated gene sets were subjected separately to a DAVID search, and only gene sets where adjusted *p* values were lower than 0.05 were selected. The DAVID software analysis showed that differently expressed genes belong to 582 gene ontology groups and 45 KEGG pathways. In this paper, we focused on the “regulation of steroid biosynthetic process” (GO:0050810) and “regulation of steroid metabolic process” (GO:0019218) GO BP terms. These sets of genes were subjected to a hierarchical clusterization procedure and presented as heat maps (Figure 1). The gene symbols, fold changes in expression, Entrez gene identifications (IDs), and corrected *p* values of these genes are shown in Table 1.

**Figure 2:**
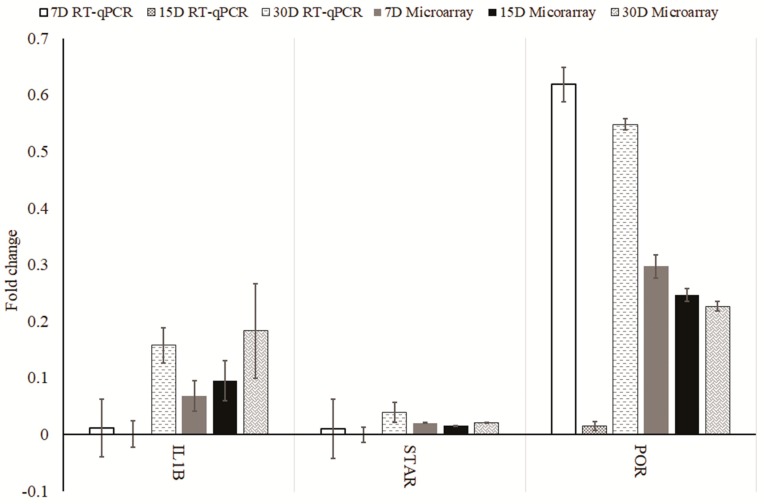
Results from RT-qPCR validation, presented in the form of a bar chart with comparisons to the results obtained with microarray. All the values presented are the relative changes of gene expression, as compared to Day 1 of primary culture. D: Day of Culture.

Additionally, using an RT-qPCR assay, we analyzed the relative abundance of *IL1B, STAR*, and *POR* in order to quantitatively validate the results obtained through the qualitative microarray analysis. The results were presented in Figure 2. *STAR* and *IL1B* were selected for validation as they presented the most extreme fold changes. *POR* was selected as a mean of control as it presented fold changes on the most intermediate levels. Additionally, *STAR* is a gene that is very important for the described steroid associated processes, while the presence of *IL1B* is especially interesting as it is usually associated with the inflammatory response and not steroid synthesis and metabolism. We have identified the presence of transcripts of the aforementioned genes in human granulosa cells.

For both *IL1B* and *STAR*, we found that the levels of transcripts at day 15 were nearly identical as at day 1. In the case of *IL1B*, this does not correspond with the microarray finding. For day 7 there was a slight change in mRNA levels observed, as compared to the entry readings. The most substantial change was observed at day 30 for both of those genes. Moreover, for both *STAR* and *IL1B*, the fold change of expression was relatively the same as in the results shown by microarray analysis, which can be considered as validation of those results. For the *POR* gene, the smallest fold change was observed at day 15, with major changes at both day 7 and 30, as compared to those at the entry point. Again, on day 15, the results of microarray and RT-qPCR analysis varied significantly in scale. Overall, the direction of changes in expression is confirmed by RT-qPCR analysis, while the scale of change often varies, which can be explained by the much higher accuracy of RT-qPCR. It does not interfere with the results of research focused on expression patterns, but indicates the need for extensive validation of microarray results during the studies involving the analysis of the extent of expression changes.

**Figure 3:**
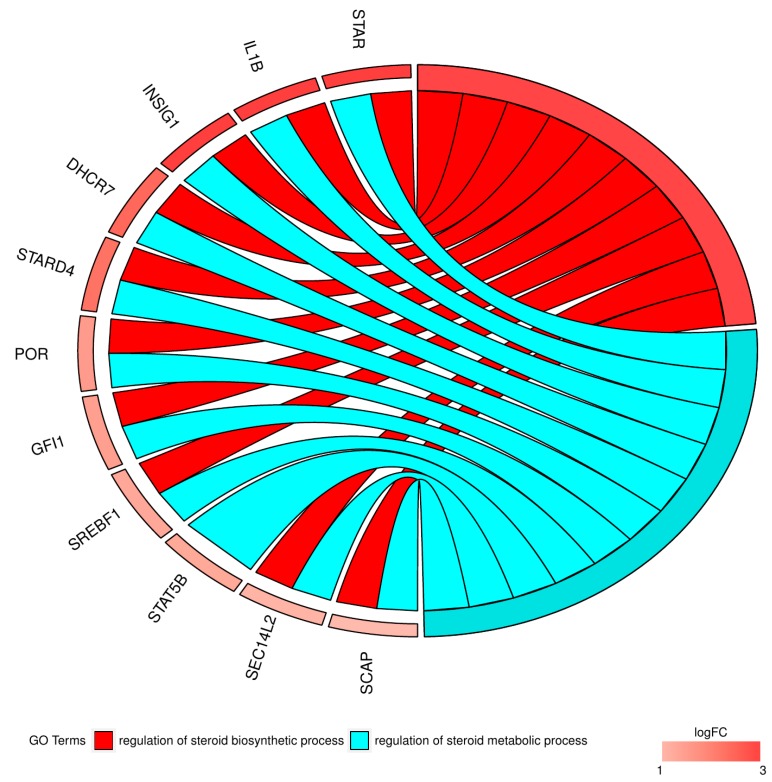
The representation of the mutual relationship between the “regulation of steroid biosynthetic process” and “regulation of steroid metabolic process” GO BP terms. The ribbons indicate which gene belongs to which categories. The genes were sorted by logFC from most to least changed gene, with the most changed gene marked with the most intense color on the side and presented topmost, and the least changed gene marked with the least intense color on the side and presented on the bottom.

Moreover, in the gene ontology database, genes that formed one particular GO group can also belong to other different GO term categories. For this reason, we explored the gene intersections between selected GO BP terms. The relation between those GO BP terms are presented in a chart (Figure 3).

**Figure 4:**
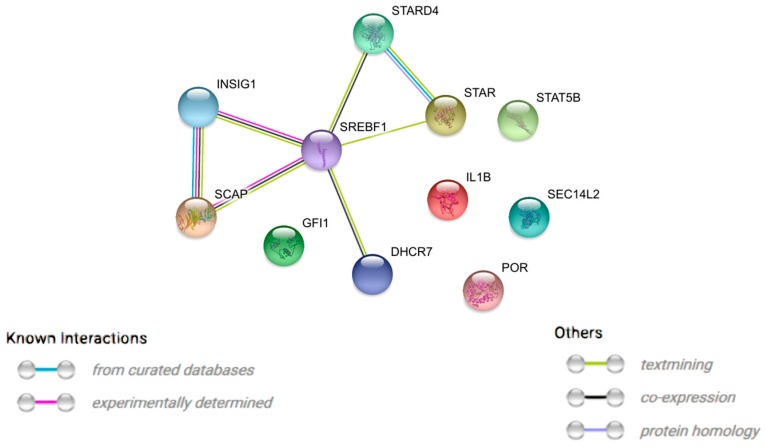
STRING (Search Tool for the Retrieval of Interacting Genes/Proteins)-generated interaction network among differentially expressed genes belonging to the “regulation of steroid biosynthetic process” and “regulation of steroid metabolic process” GO BP terms. The intensity of the edges reflects the strength of the interaction score.

We used a STRING (Search Tool for the Retrieval of Interacting Genes/Proteins)-generated network to evaluate the interactions between differentially expressed genes belonging to each of the selected GO BP terms. Using this prediction method provided us with the molecular interaction network which is formed between the protein products of the studied genes (Figure 4).

## 3. Discussion

Granulosa cells are the primary cell type found in the mammalian ovary. They perform multiple functions associated with oocyte storage, maturation, as well as maintaining pregnancy and embryonic development. Because of that, it can be said that they are a somatic cell type that carries the strongest and closest association with the processes underlying development of female gametes and their progression through the process of reproduction. They surround the oocyte all the way from the primary follicle to the moment of ovulation, performing a major role in synthesis and expression of sex hormones [1,10]. The interactions between oocyte and granulosa cells are reciprocal, with both structures expressing signals that influence the development and functioning of the other. After the follicular rupture, granulosa cells that remain in the newly formed corpus luteum undergo transition into granulosa lutein cells, which produce progesterone, taking further part in maintenance of pregnancy. While the function of granulosa cells is fairly well understood, the exact mechanisms and genes expressed through the significant amount of change undergone by these cell types is still full of unknowns [1]. Because of their major significance for the process of follicle development and oogenesis, the complete understanding of gene expression underlining the functioning of granulosa cells and their interactions with oocytes is essential for developing possible clinical applications associated with gene therapy, tissue and cell engineering, as well as prevention, early detection, and treatment of cancers originating in the ovarian region that associated with granulosa cells [11].

By using the microarray approach, we have identified and measured the changes in expression of genes that are part of ontological groups of interest. The RNA analyzed was isolated from primary cell cultures of granulosa cells after 1, 7, 15, and 30 days of maturation in vitro. Two gene ontology groups were inspected: (1) “Regulation of steroid biosynthesis process” and (2) “Regulation of steroid metabolic process”. Eleven genes were identified, out of which ten, namely *STAR, SCAP, POR, SREBF1, GFI1, SEC14L2, STARD4, INSIG1, DHCR7* and *IL1B*, belonged to both groups. One of the genes, *STAT5B*, only belonged to the “Regulation of Steroid Metabolic Process” ontological group. Most of the genes presented a uniform pattern of expression. Large upregulation of expression was observed at day 1 of cell culture, with significant downregulation at days 7, 15, and 30. Three genes showed a different pattern at day 30: *INSIG1* and *DHCR7* had very little downregulation and *IL1B* had no change in expression. Despite that, we can assume that the pattern of expression was fairly unified for all the genes, with upregulation at day 1 and general downregulation through the other days. There are several readings that could indicate a slight upregulation in expression in several genes in different days of cell cultures apart from day 1, however, all of the readings do not appear consistently in both reads performed and are contradicted by strong downregulation recorded in the other read, which indicates that they are probably no more than an error in measurement.

Two of the genes identified are very closely associated with processes underlying steroid intracellular transport. They are both hosts to the *STAR*-related transfer domain (START), with the main function of binding lipids, including sterols [12]. STAR steroid acute regulatory protein, otherwise called *STARD1*, encodes a protein that is a key factor in regulation of steroid hormone synthesis. By mediating the transport of cholesterol between mitochondrial membranes, the protein allows cholesterol to be converted into pregnenolone, completing the first major step in the process of steroidogenesis [13,14]. Disruption of the *STAR* gene has been proven to cause congenital lipoid adrenal hyperplasia (CLAH) [15], an endocrine disorder, causing mineralocorticoid deficiency which impairs the synthesis of all categories of adrenal steroids [16]. The other gene that hosts the START domain is the *STAR*-related lipid transfer protein 4 (*STARD4*). It is a gene encoding a soluble protein involved in cholesterol transport. It is a cholesterol regulated gene with two known homologues belonging in its family: *STARD5* and *STARD6*. While being expressed in most tissues, the highest levels of *STARD4* are observed in the liver and kidneys [17]. There are strong suggestions of *STARD4*’s particular involvement in movement of cholesterol to the endoplasmic reticulum [18].

Three of the genes identified work closely for maintenance of intracellular cholesterol homeostasis. The sterol regulatory element binding transcription factor 1 (*SREBF1*) gene encodes a basic helix-loop-helix leucine zipper transcription factor (*SREBP1*) binding the sterol regulatory element (SRE1) [19]. *SREBP1* is stored as a membrane bound precursor and is released by proteolytic cleavage in sterol depleted cells. The loose fragment generated through the cleavage translocates to the nucleus, activating transcription [20]. It has been proven to play a major role in the induction of lipogenesis, as well as an auxiliary factor in synthesis of fatty-acids [21]. *SCAP* is closely associated with *SREBP1*, the protein encoded by *SREBF1*. It is an escort protein that regulates the effects of *SREBP1* expression, detecting the changes of cholesterol concentration. It is the effector of *SREBP1* cleavage, allowing that transcription factor to abandon its membrane-bound state, transduce to the nucleus, and allow transcription of the genes activated by the SREBP1 [22]. The cleavage occurs in the Golgi apparatus, which requires *SCAP* to escort *SREBP1* to the Golgi apparatus from the endoplasmic reticulum, where *SREBP1* is synthesized [23]. *SREBP1* transcriptional regulation effects also cause negative feedback by causing degradation of *SCAP* to prevent sterol overexpression [24]. Large concentrations of sterols prevent the exit of the *SREBP1*/*SCAP* complex from the endoplasmic reticulum in order to prevent accumulation of cholesterol inside of the cell [22]. This regulative step is achieved with involvement of another gene, insulin induced gene 1 (*INSIG1*). This gene encodes a membrane protein found in the endoplasmic reticulum. In the presence of high sterol concentrations, *INSIG1* binds the sterol-sensing domain of *SCAP*. This causes retention of the *SREBP1*/*SCAP* complex in the endoplasmic reticulum and prevents its translocation to the Golgi apparatus, therefore, prevents cleavage of *SREBP1* and cholesterol synthesis upregulation [25].

The genes described above are closely associated in the process involved in lipid synthesis and metabolism. However, there are several more genes that were identified in our study that possess individual involvement with lipid metabolic and synthetic processes. *POR* is a gene encoding cytochrome P450 oxidoreductase. This enzyme plays a critical role in the synthesis of cholesterol and steroid hormones as an obligatory intermediate which transfers electrons from NADPH (nicotinamide adenine dinucleotide phosphate carrying electrons and bonded with a hydrogen) to all cytochrome P450 enzymes [26]. It is also involved in the metabolism of ingested substances in the liver, and it has been suggested that *POR* gene polymorphisms are involved in differences in drug metabolism across the population [27]. Defects in *POR* genes have been proven to cause a variety of symptoms, from embryonic lethality to disordered steroidogenesis, congenital adrenal hyperplasia, ambiguous genitalia, and Antley-Bixler syndrome [26,28,29]. The next gene, *DHCR7*, encodes an enzyme, 7-dehydrocholesterol reductase, that catalyzes the conversion of 7-dehydrocholesterol to cholesterol. This fact, makes the enzyme essential in mammalian sterol biosynthesis, as it is necessary in the final step of the synthesis pathway [30]. Defects in *DHCR7* gene were proven to be the cause of Smith-Lemli-Opitz syndrome, which may cause mental retardation, dysmorphism of the face, syndactyly, and holoprosencephaly as a result of insufficient sterol synthesis and 7-dehydrocholesterol accumulation [30,31]. The *SEC14L2* gene encodes SEC14 like protein 2. This lipid-binding carrier protein facilitates transport of hydrophobic molecules between different cellular sites [32]. *SEC14L2* exhibits high affinity to alpha-tocopherol and weaker affinity to other tocopherols and tocotrienols. It has been proven to stimulate squalene monooxygenase, a downstream enzyme in cholesterol biosynthesis [33]. *IL-1B* encodes an interleukin 1 cytokine family member protein, which functions as an important mediator of the inflammatory response [34]. It is usually expressed by activated macrophages in its inactive form, which is later activated by proteolytical processing [35]. The cytokine encoded by *IL-1B* is also involved in other cellular processes including proliferation, differentiation, and apoptosis [36,37,38]. Proto-oncogene *GFI1* (growth factor independent 1 transcriptional repressor) encodes a nuclear zinc finger protein, functioning as a transcriptional repressor. The repression is achieved by *GFI1* working with other cofactors as a part of a complex that controls histone modifications and silences target gene promoters [39]. It has been proven to play a role in processes such as hematopoiesis and oncogenesis [40]. Mutations in that gene can cause neutropenia in both the congenital and nonimmune chronic idiopathic forms, both being autosomal dominant disorders causing predispositions to leukemia and infections [41]. The final gene described in this study is *STAT5B*, signal transducer and activator of transcription 5B. This gene is only a member of the “Regulation of steroid metabolic process” gene ontology group. It encodes a STAT family transcription factor, expression of which is activated by cytokine activity. Transcription factors belonging to that family function when phosphorylated by the receptor associated kinases. After phosphorylation, hetero or homodimerization occurs, with the resulting complex functioning as a transcription activator after translocating to the nucleus [42]. *STAT5B* performs various functions, mostly associated with hematopoiesis, mammary gland development, and immune systems [43,44,45].

In conclusion, we identified and described the genes expressed in long term in vitro cultures, belonging to the ontology groups “Regulation of steroid biosynthesis process” and “Regulation of steroids metabolic process”. Analysis of the modes of their expression, together with their relation to each other, will broaden the understanding of the ways in which the cells of human granulosa are involved in the processes associated with steroid synthesis and metabolism, which may later be applied in both further research and, potentially, together with other research that improves the understanding of the molecular basis of this tissue’s functioning, to clinical applications.

## 4. Material and Methods

### 4.1. Patients and Collection of Granulosa Cells

The granulosa cells (GCs) were derived from patients undergoing in vitro fertilization (IVF) procedures, who had given their informed consent to be included in this protocol. The study group consisted of eight patients, aged 18–40 years, with diagnosed infertility who were referred to the Division of Infertility and Reproductive Endocrinology, Poznan University of Medical Sciences, Poland. Patients underwent an IVF procedure based on controlled the ovarian hyperstimulation protocol, adjusted to the patient’s initial infertility workup and ovarian response. Stimulation was performed with human recombinant FSH (Follicle-stimulating hormone; Gonal-F, Merck Serono, Darmstadt, Germany) and highly purified hMG-HP (Highly purified human menopausal gonadotropin; Menopur, Ferring, Saint-Prex, Switzerland). The injections with cetrorelix acetate (Cetrotide, Merck Serono, Darmstadt, Germany) were administered in an adequate dose to suppress pituitary function. Ovulation triggering was based on subcutaneous injection of 6500 U of hCG (Human chorionic gonadotropin, Ovitrelle; Merck-Serono, Darmstadt, Germany). Follicular fluid, containing GCs, was collected during transvaginal ultrasound-guided oocyte pick-up, 36 h after human chorionic gonadotropin administration. The follicular content from follicles over 16 mm in diameter was given rapidly to the embryologist, who isolated the oocyte and pooled the fluids containing GCs together from each ovary. Fresh follicular fluid was centrifuged for 10 min at 200× *g*, to separate and collect GCs. Patients with polycystic ovary syndrome (PCOS), endometriosis, and diminished ovarian reserve (serum antimüllerian hormone (AMH) less than 0.7 ng/mL and/or day 2–3 FSH serum level higher than 15 mU/mL and/or antral follicle count less than 9) were excluded from the study. This study was approved with resolution 558/17 by the Bioethical Committee at the Poznan University of Medical Sciences. All participants gave written informed consent for the research.

### 4.2. Primary Cell Culture

**Figure 5:**
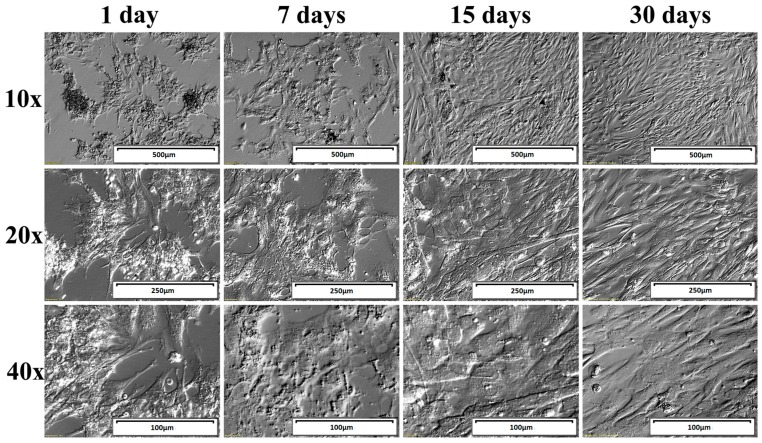
Morphology of human ovarian granulosa cells in long term in vitro culture shown using Nomarski phase/contrast images.

Collected cells were washed twice by centrifugation at 200× *g* for 10 min at room temperature with culture medium. Medium consisted of Dulbecco’s Modified Eagle’s Medium (DMEM/F12, Sigma-Aldrich, St. Louis, MO, USA), 2% fetal bovine serum FBS (FBS; Sigma-Aldrich Co., St. Louis, MO, USA), 200 mM l-glutamine (Invitrogen, Carlsbad, CA, USA), 10 mg/mL gentamicin (Invitrogen, USA), 10,000 units/mL penicillin, and 10,000 μg/mL streptomycin (Invitrogen, USA). Cells were cultivated at 37 °C under aerobic conditions (5% CO_2_). Once adherent cells were more than 90% confluent, they were detached with 0.05% trypsin-EDTA (Invitrogen, USA) for 1–2 min and counted using a counting chamber “neubauer improved” (ISO LAB Laborgerate GmbH, Wertheim, Germany, DIN EN ISO CERTIFIED 9001). GCs were cultivated for 30 days. Medium was changed twice a week. Finally, total RNA was isolated from GCs after 24 h, 168 h, 15 days, and 30 days. The changes in cell morphology were presented in Figure 5.

### 4.3. Total RNA Isolation

Total RNA was isolated at four time periods, after 24 h, 168 h, 15 days, and 30 days cultivation. For the isolation of total RNA, we used the improved Chomczyński-Sacchi method [46]. The GCs were suspended in 1 mL mixture of guanidine thiocyanate and phenol in monophasic solution (TRI Reagent^®^, Sigma-Aldrich, St. Luis, MO, USA). Then, chloroform was added and centrifuged to separate the mixture into three phases. RNA was located in the upper phase—an aqueous phase. The resulting RNA was intact with little or no contaminating DNA and protein. The last step was to strip the RNA with 2-propanol (Sigma-Aldrich, St. Luis, MO, catalog number I9516) per 1 mL of TRI-reagent and wash with 75% ethanol. Prepared RNA was used for further analysis.

### 4.4. Microarray Expression Analysis and Statistics

Total RNA (100 ng) from each pooled sample was subjected to two rounds of sense complementary DNA (cDNA) amplification (Ambion^®^ WT Expression Kit; Ambion, Austin, TX, USA). The obtained cDNA was used for biotin labeling and fragmentation by Affymetrix GeneChip^®^ WT Terminal Labeling and Hybridization (Affymetrix, Santa Clara, CA, USA). Biotin-labeled fragments of cDNA (5.5 μg) were hybridized to the Affymetrix^®^ Human Genome U219 Array (48 °C/20 h; Affymetrix, Santa Clara, CA, USA). Microarrays were then washed and stained according to the technical protocol using the Affymetrix GeneAtlas Fluidics Station. The array strips were scanned employing the Imaging Station of the GeneAtlas System. Preliminary analysis of the scanned chips was performed using Affymetrix GeneAtlasTM Operating Software (Affymetrix, Santa Clara, CA, USA). The quality of gene expression data was confirmed according to the quality control criteria provided by the software. The obtained CEL files were imported into downstream data analysis software.

All of the presented analyses and graphs were performed using Bioconductor and R programming languages. Each CEL file was merged with a description file. In order to correct background, normalize, and summarize results, we used the Robust Multiarray Averaging (RMA) algorithm. To determine the statistical significance of the analyzed genes, moderated t-statistics from the empirical Bayes method were performed. The obtained *p*-value was corrected for multiple comparisons using Benjamini and Hochberg's false discovery rate. The selection of significantly altered genes was based on a *p*-value beneath 0.05 and expression higher than two-fold. The differentially expressed gene list (separated for up- and down-regulated genes) was uploaded to DAVID (Database for Annotation, Visualization and Integrated Discovery) software (Leidos Biomedical Research, Inc., National Cancer Institute, Frederick, MD, USA) [47].

Subsequently, sets of differentially expressed genes from selected GO BP terms were applied to STRING software (Search Tool for the Retrieval of Interacting Genes/Proteins; STRING Consortium) for interaction predictions. STRING is a huge database containing information of protein/gene interactions, including experimental data, computational prediction methods, and public text collections.

In order to further investigate the chosen gene sets, we investigated its mutual relations with the GOplot package [48]. Moreover, the GOplot package allowed us to calculate the z-score (the number of up- regulated genes minus the number of down- regulated genes divided by the square root of the count). Z-score analysis allowed us to compare the enrichment of selected GO BP terms.

### 4.5. Real-Time Quantitative Polymerase Chain Reaction (RT-qPCR) Analysis

Total RNA was isolated from granulosa cells after 24 h, 7 days, 15 days, and 30 days of culture. The RNA samples were re-suspended in 20 μL of RNase-free water and stored in liquid nitrogen. RNA samples were treated with DNase I and reverse-transcribed (RT) into cDNA. RQ-PCR was conducted in a LightCycler real-time PCR detection system (Roche Diagnostics GmbH, Mannheim, Germany) using SYBR^®^ Green I as a detection dye, and target cDNA was quantified using the relative quantification method. For amplification, 2 μL of cDNA solution was added to 18 μL of QuantiTect^®^ SYBR^®^ Green PCR (Master Mix Qiagen GmbH, Hilden, Germany) and primers (Table 2).

One RNA sample of each preparation was processed without the RT-reaction to provide a negative control for subsequent PCR.

To quantify specific genes in the granulosa cells, expression levels of specific messenger RNAs (mRNAs) were calculated relative to *GAPDH* (Glyceraldehyde-3-Phosphate Dehydrogenase), *HPRT* (Hypoxanthine guanine phosphoribosyltransferase), and *ACTB* (Beta-actin). To ensure the integrity of these results, the additional housekeeping gene was used as an internal standard to demonstrate that *GAPDH, HPRT*, and *ACTB* mRNAs were not differentially regulated in the granulosa cells.

## Figures and Tables

**Table 1:** Gene symbols, fold changes in expression, Entrez gene identifications (IDs), and corrected *p* values of studied genes. Adjusted *p* value = adj.P.Val.

Official Gene Symbol	Fold Change D1/D7	Fold Change D1/D15	Fold Change D1/D30	adj.P.Val. D1/D7	adj.P.Val. D1/D15	adj.P.Val. D1/D30	Entrez Gene ID
*DHCR7*	0.174701154	0.135435372	0.287884941	0.039383847	0.023551624	0.084599974	1717
*GFI1*	0.310857826	0.318291331	0.286701236	0.001572194	0.001472925	0.000931728	2672
*IL1B*	0.068991209	0.095213232	0.183379317	0.027149434	0.035799475	0.083315453	3553
*INSIG1*	0.112401766	0.111379044	0.231549573	0.040421878	0.035709593	0.102319923	3638
*POR*	0.297205384	0.246609697	0.226675727	0.02007617	0.011465695	0.008491922	5447
*SCAP*	0.428465981	0.37245418	0.350801297	0.00234869	0.001405483	0.000934051	22937
*SEC14L2*	0.393655866	0.47190842	0.474492018	0.049169113	0.080230709	0.075670468	23541
*SREBF1*	0.342373023	0.40104925	0.351769946	0.004735431	0.007059315	0.004002643	6720
*STAR*	0.020791115	0.015709262	0.021002203	0.000945846	0.000687578	0.000705428	6770
*STARD4*	0.192889636	0.155104665	0.215393387	0.037708661	0.023368014	0.037965777	134429
*STAT5B*	0.343238914	0.381840346	0.358794257	0.008451029	0.010289799	0.007445427	6777

**Table 2:** Oligonucleotide sequences of primers used for RT-qPCR analysis.

Gene	Gene Accession Number	Primer Sequence (5′-3′)	Product Size (bp)
*STAR*	NM_000349.2	GGCATCCTTAGCAACCAAGA TCTCCTTGACATTGGGGTTC	199
*Il1B*	NM_000576.2	GGGCCTCAAGGAAAAGAATC TTCTGCTTGAGAGGTGCTGA	205
*POR*	NM_000941.2	CACAAGGTCTACGTCCAGCA GCCACGATGTCGTAGAAGGT	143
*GAPDH*	NM_002046	TCAGCCGCATCTTCTTTTGC ACGACCAAATCCGTTGACTC	90
*ACTB*	NM_001101	AAAGACCTGTACGCCAACAC CTCAGGAGGAGCAATGATCTTG	132
*HPRT*	NM_000194	TGGCGTCGTGATTAGTGATG ACATCTCGAGCAAGACGTTC	141
